# Homer1 promotes the conversion of A1 astrocytes to A2 astrocytes and improves the recovery of transgenic mice after intracerebral hemorrhage

**DOI:** 10.1186/s12974-022-02428-8

**Published:** 2022-03-14

**Authors:** Xiaowei Fei, Ya-nan Dou, Li Wang, Xiuquan Wu, Yu Huan, Shuang Wu, Xin He, Weihao Lv, Jialiang Wei, Zhou Fei

**Affiliations:** Department of Neurosurgery, Xijing Hospital, Air Force Military Medical University, No. 127, Changle West Road, Xincheng District, Xi’an, 710032 Shaanxi China

**Keywords:** Homer1, Intracerebral hemorrhage, Inflammation, Astrocytes, Phenotype

## Abstract

**Background:**

Inflammation induced by intracerebral hemorrhage (ICH) is one of the main causes of the high mortality and poor prognosis of patients with ICH. A1 astrocytes are closely associated with neuroinflammation and neurotoxicity, whereas A2 astrocytes are neuroprotective. Homer scaffolding protein 1 (Homer1) plays a protective role in ischemic encephalopathy and neurodegenerative diseases. However, the role of Homer1 in ICH-induced inflammation and the effect of Homer1 on the phenotypic conversion of astrocytes remain unknown.

**Methods:**

Femoral artery autologous blood from C57BL/6 mice was used to create an ICH model. We use the A1 phenotype marker C3 and A2 phenotype marker S100A10 to detect astrocyte conversion after ICH. Homer1 overexpression/knock-down mice were constructed by adeno-associated virus (AAV) infection to explore the role of Homer1 and its mechanism of action after ICH. Finally, Homer1 protein and selumetinib were injected into in situ hemorrhage sites in the brains of Homer1^flox/flox^/Nestin-Cre^+/−^ mice to study the efficacy of Homer1 in the treatment of ICH by using a mouse cytokine array to explore the potential mechanism.

**Results:**

The expression of Homer1 peaked on the third day after ICH and colocalized with astrocytes. Homer1 promotes A1 phenotypic conversion in astrocytes in vivo and in vitro. Overexpression of Homer1 inhibits the activation of MAPK signaling, whereas Homer1 knock-down increases the expression of pathway-related proteins. The Homer1 protein and selumetinib, a non-ATP competitive MEK1/2 inhibitor, improved the outcome in ICH in Homer1^flox/flox^/Nestin-Cre^+/−^ mice. The efficacy of Homer1 in the treatment of ICH is associated with reduced expression of the inflammatory factor TNFSF10 and increased expression of the anti-inflammatory factors activin A, persephin, and TWEAK.

**Conclusions:**

Homer1 plays an important role in inhibiting inflammation after ICH by suppressing the A1 phenotype conversion in astrocytes. In situ injection of Homer1 protein may be a novel and effective method for the treatment of inflammation after ICH.

## Background

Astrocytes are the most abundant cell type in the central nervous system and have a wide range of functions, such as in transsynaptic signal transmission [1], potassium ion spatial buffering [2], maintenance of environmental homeostasis [3], and transmission of immune signals [4, 5]. Similar to the two adaptive states M1 and M2 of macrophages, two phenotypes, A1 and A2, are also present in astrocytes [6–8]. During inflammation, bacterial lipopolysaccharide induces astrocytes to be converted to the deleterious A1 phenotype [9], releasing neurotoxic factors such as complement components and inflammatory cytokines to mediate neuronal and oligodendrocyte cell death [6, 10–12]. Hypoxia induces A2 astrocytes, which exert neuroprotective functions by producing anti-inflammatory cytokines and neurotrophic factors [6, 12–14]. In addition, A1 astrocytes specifically express complement C3, whereas A2 astrocytes specifically express S100A10, which is an important biomarker for distinguishing A1 and A2 astrocytes [6, 15]. Intracerebral hemorrhage (ICH) triggers astrocyte reactivity, which regulates the release of pro-inflammatory and anti-inflammatory factors [16, 17]. Dynamic polarization of reactive A1 and A2 astrocytes occurs during the pathological process of ICH, ranging from days to weeks. However, the temporal and spatial transitions of these two astrocyte states and key regulators of astrocyte reactivity after ICH remain unclear.

Homer scaffold protein 1 (Homer1) is a postsynaptic density scaffold protein composed of an EVH protein binding domain, a coiled-coil domain, and a leucine zipper domain [18, 19]. Our previous findings suggested that Homer1 knock-down protects dopamine neurons by modulating calcium homeostasis in an in vitro model of Parkinson's disease [20]. It has also been reported that oxidative stress and inflammation of cerebral microvessels caused by hypertension are related to downregulation of Homer1 [21]. Neuropathological processes after ICH are associated with cellular stress by influencing inflammatory activation, reactive gliosis, and neuronal survival [22, 23]. Although Homer1 is dynamically regulated in inflammatory responses, its precise role in the regulation of cellular events has not been fully revealed. Given that endogenous Homer1 is sufficient to inhibit the expression of inflammatory cytokines TNF-α and IL-1β [24], hallmarks of A1 astrocyte secretion, it was hypothesized that Homer1 may control the reactive astrocyte state after ICH.

In this study, the relationship between spatiotemporal expression of Homer1 and the reactive astrocyte status after ICH was analyzed in mice. Mitogen-activated protein kinase (MAPK) signaling pathways implicated in reactive astrocyte activation were also investigated. We show that Homer1 effectively inhibits A1 phenotypic conversion of astrocytes after ICH in mice, and in situ treatment with Homer1 protein improves the outcome in Homer1^flox/flox^/Nestin-Cre^+/−^ mice after ICH.

## Methods

### Animals

All animal experiments were performed in accordance with protocols approved by the Institutional Ethics Committee of Xijing Hospital. All experimental procedures were approved by the Institutional Animal Care and Use Committee of Air Force Military Medical University. C57BL/6 and Homer1^flox/flox^/Nestin-Cre^+/−^ mice were purchased from the Shanghai Model Organisms Center, Inc. (Shanghai, China). All mice were maintained in the same environment.

### Lentivirus and adeno-associated virus

Primary astrocytes were infected with lentivirus to stably overexpress Homer1 (Homer1-OE) (LV-hU6-Homer1-Ubiquitin-EGFP-IRES-puromycin) or knock-down Homer1 (Homer1-KD) (LV-Ubi-shRNAHomer1-3FLAG-SV40-EGFP-IRES-puromycin). The lentivirus was constructed with the assistance of GeneChem Co., Inc. (Shanghai, China).

Mice were infected with adeno-associated virus (AAV) to stably overexpress Homer1 (AAV2/9-CMV-Homer1**)** or knock-down Homer1 (AAV2/9-U6-shRNAHomer1-WPRE) at the ICH site. The AAVs were constructed with the assistance of Hanbio Co., Inc. (Shanghai, China). The targeting sequence of the siRNA was 5′-GCATTGCCATTTCCACATA-3 and the transcript sequence for overexpressing Homer1 was NM_011982.

### ICH model

The ICH model was established as previously described with minor modifications [25, 26]. The mice were anesthetized with 4% chloral hydrate (400 mg/kg) injected intraperitoneally. The rectal temperature was maintained at 37.5 °C. A stereotactic technique was used to make a scalp incision along the midline and a burr hole was drilled on the left side of the skull (0.2 mm anterior and 2.5 mm lateral to the bregma). Thirty microliters of autologous blood obtained from the femoral artery were transferred into a 50 μL Hamilton syringe. The syringe was connected to a microinjection pump and the needle was inserted into the brain through the burr hole (depth, 3.5 mm from the bone surface). Thirty microliters of autologous blood were injected within 10 min. The syringe was withdrawn after 10 min, and selumetinib (MCE, AZD6244) and Homer1 protein (EUPROTEIN, EP8767430) (5 mg/kg, dissolved in normal saline) were re-injected in situ for 10 min. After surgery, the skull hole was sealed with bone wax and the incision was closed with sutures. To avoid postsurgical dehydration, normal saline (0.5 mL) was subcutaneously injected into each mouse immediately after the surgery. The ICH model mice were killed at different time points for staining and western blot (WB) analyses.

### Experimental grouping

Ten C57BL/6 mice were used as the sham group. Thirty C57BL/6 mice were used to establish the ICH model, and five mice per group were anesthetized on days 1, 3, and 7. The brain tissue around the hemorrhage site in each mouse was extracted and stored at  80 °C for WB, quantitative real-time polymerase chain reaction (qPCR), and enzyme-linked immunosorbent assay (ELISA) analyses. Each experiment was repeated three times (*n* = 5/group). In addition, five mice in each group were anesthetized and killed to obtain frozen sections. The frozen sections with the largest bleeding area in each mouse were selected for immunofluorescence stainings. One field at the same magnification was randomly selected from one section of each mouse for positive cell counting and quantification (*n* = 5/group). Mice were scored according to the Longa score standard before anesthesia in all groups (*n* = 10/group).

Three weeks before ICH modeling, AAV was injected into the cerebral hemorrhage site under the same coordinates, and ten Homer1-OE and ten Homer1-KD mice were generated. AAV was stably expressed after 3 weeks of normal feeding. Ten C57BL/6 mice were used in the sham group (Sham group). Ten ordinary C57BL/6 mice (ICH group), 10 Homer1-OE mice (Homer1-OE group), and 10 Homer1-KD mice (Homer1-KD group) were used to establish the ICH model. On the third day after ICH, all mice were anesthetized and killed. The hemorrhagic brain tissue of five mice in each group was used for WB, qPCR, and ELISA assays (*n* = 5/group). The brain tissue of five mice in each group was used to obtain frozen sections and for immunohistochemistry (*n* = 5/group). Mice were scored according to the Longa score standard before anesthesia in all groups (*n* = 10/group). In addition, 20 mice from each group were used for survival analysis (*n* = 20/group).

Fifty Homer1^flox/flox^/Nestin-Cre^+/−^ mice were divided into five groups: sham, ICH, Homer1 protein, selumetinib, and Homer1 + selumetinib group (Both). ICH models were established in all groups, except the sham group, and different drugs were administered according to the design. On the third day after ICH, all mice were anesthetized and killed. The hemorrhagic brain tissue of five mice in each group was used for WB, qPCR, and ELISA assays (*n* = 5/group), and mouse cytokine array detection (*n* = 3/group). The brain tissue of five mice was used to obtain frozen sections and for immunohistochemistry (*n* = 5/group). The mice were scored according to the Longa score standard before anesthesia in all groups (*n* = 10/group). In addition, 20 Homer1^flox/flox^/Nestin-Cre^+/−^ mice in each group were used for survival analysis.

### Cell culture and treatment

Neonatal C57 mice were used to extract primary astrocytes. The brain tissue was minced with sterile ophthalmic scissors, digested with 0.25% trypsin for 5 min at 37 °C before the brain tissue was centrifuged at 1000 rpm for 5 min. The digested tissue was cultured in F-12 medium enriched with 10% fetal bovine serum (FBS), 0.224% NaHCO_2_, and 1% penicillin/streptomycin at 37 °C in the presence of 5% CO_2_. Astrocyte monolayers were obtained at the bottom of the dish after 2 weeks. The astrocytes were identified by morphological analysis and GFAP staining. Before further treatment, the medium containing 0.5% FBS was replaced to ensure that the cells were in a resting state.

The ICH cell model was established as previously described [27]. Cells were treated with erythrocyte lysate (1 μL of red blood cell lysate per mL of medium) to create an in vitro ICH inflammation model. The cells were incubated for different durations and used in different experiments. Erythrocyte lysates were prepared with red blood cell lysis buffer (Solarbio, Beijing), and the experimental process was in strict accordance with the manufacturer’s instructions.

A1 astrocytes were generated as described by Liddelow et al. [6]. Astrocyte conditional medium (ACM) was prepared in DMEM supplemented with TNF-α (30 ng/mL, CST, 8902), IL-1α (3 ng/mL, Peprotech, 400-01), and C1q (400 ng/mL, Novus protein, NBP2-62410). Conversion of astrocytes from A1 to A2 was examined by protein and mRNA expression of C3 and S100A10 after the cells were cultured in ACM for 24 h.

### qPCR

Primary astrocytes were harvested for RNA extraction after different treatments using TRIzol reagent. Mice were anesthetized at different time points after ICH induction, and the brain tissue around the bleeding site was used for qPCR. Reverse transcription was performed according to the protocol of the HiScript II Q Select RT SuperMix for qPCR (+ gDNA wiper) kit (Vazyme, China). qPCR was performed according to the protocol of the ChamQ SYBR Color qPCR Master Mix (Low ROX Premixed) kit (Vazyme, China). The primers for mRNA were as follows: Homer1-F: “AATGGTTAGGGGGCACTGTTT,” Homer1-R: “CCCATCTGCCACAGTCACAA”; C3-F: “AACAAGCTCTGCCGTGATGA,” C3-R: “GCCTGACTTGATGGTCTGCT”; S100A10-F: “TGAGAGTGCTCATGGAACGG,” S100A10-R: “AGAAAGCTCTGGAAGCCCAC”; GAPDH-F: “AGAGGCCCTATCCCAACTCG,” and GAPDH-R: “GTGGGTGCAGCGAACTTTATT”. The expression of related RNAs was calculated using the 2^−ΔΔCt^ method, and GAPDH was used as a control. The experiment was repeated thrice.

### Western blot

Western blotting was performed as previously described [25]. Primary astrocytes were harvested for protein extraction after the different treatments. Mice were anesthetized at different time points after ICH induction, and the brain tissue around the bleeding site was used for WB. The following antibodies were used: rabbit anti-Homer1 (1:1000; Abcam), rabbit anti-C3 (1:1000; Abcam), rabbit anti-S100A10 (1:1000; Proteintech), mouse anti-GAPDH (1:10,000; Abcam), rabbit anti-Ras (1:1000; CST), rabbit anti-Phospho-c-Raf (Ser338) (1:1000; CST), mouse anti-Raf-1 (1:1000; Proteintech), rabbit anti-Phospho-MEK1/2 (Ser217/221) (1:1000; CST), mouse anti-MEK1/2 (1:1000; CST), rabbit anti-Phospho-ERK1/2 (Thr202/Tyr204) (1:1000; Proteintech), rabbit anti-ERK1/2 (1:1000; Proteintech), and goat anti-mouse/rabbit secondary antibody (1:10,000; Abcam).

### Nissl staining

Frozen sections with the largest bleeding areas were selected for Nissl staining. The sections were washed with PBS three times for 5 min each time and stained with 1% toluidine blue (Beyotime, Shanghai) at 56 °C for 20 min. The slices were washed with PBS three times for 5 min each. The slices were soaked in 75% alcohol for 1 min, differentiated in 95% alcohol, and the degree of differentiation was observed under a microscope. The sections were then dehydrated in absolute ethanol and soaked in xylene for 5 min. After sealing with neutral resin, the samples were observed under an optical microscope.

### TUNEL staining

Frozen sections with the largest areas of bleeding were selected for TUNEL staining. Experimental procedures for TUNEL staining were performed in strict accordance with the manufacturer’s instructions (Beyotime, Shanghai, China). Stained sections were observed under a fluorescence microscope. The excitation and emission wavelengths were 550 nm and 570 nm, respectively.

### Immunohistochemistry

Immunohistochemical analysis was performed as previously described [25]. Mice were anesthetized at different time points after ICH induction, and frozen sections with the largest bleeding area were selected for immunofluorescence analysis. The following antibodies were used: mouse anti-GFAP (1:300; CST), mouse anti-Iba1 (1:250; GeneTex), mouse anti-NeuN (1:300; CST), rabbit anti-Homer1 (1:200; Abcam), rabbit anti-C3 (1:200; Abcam), rabbit anti-S100A10 (1:300; Proteintech), donkey anti-rabbit IgG (H + L) highly cross-adsorbed secondary antibody, Alexa Fluor Plus 488 (1:1000; Invitrogen), and donkey anti-mouse IgG (H + L) highly cross-adsorbed secondary antibody, Alexa Fluor Plus 555 (1:1000; Invitrogen).

### ELISA

Cell supernatants from the different treatment groups were harvested for ELISA. Mice were anesthetized at different time points after ICH induction, and the brain tissue around the bleeding site was used for ELISA. ELISA was performed in strict accordance with the manufacturer’s instructions. The following ELISA kits were used for detection: Mouse IL-1 beta ELISA Kit (Abcam, UK), Mouse TNF alpha ELISA Kit (Abcam, UK), Mouse Tweak ELISA Kit (Abcam, UK), Mouse Activin A ELISA Kit (Abcam, UK), Mouse TRAIL ELISA Kit (Abcam, UK), and Mouse Persephin (Pspn) ELISA Kit (4A Biotech, China).

### Mouse cytokine array

The brain tissue around the bleeding site was used for the detection of inflammatory factors and cytokines. The mouse cytokine array Q4000 was purchased from RayBiotech (QAM-CAA-400; USA). The experimental procedures were performed in strict accordance with the manufacturer’s instructions.

### Mouse genotype detection

Mouse tails were cut, digested with proteinase K for 20 min at 55 °C, and further inactivated with proteinase K for 5 min at 100 °C. Polymerase chain reaction (PCR) was performed according to the protocol of the One Step Mouse Genotyping Kit (Vazyme, China). Identification of Homer1 Flox (wild type: 232 bp; mutant: 300 bp): Primer 1 (Forward): 5′-TGAGCTGGACACCCCCTGCC-3′, Primer 2 (Reverse): 5′- TGTTAAAACAATTACACCCGATTCTT -3′; identification of Nestin-Cre (wild type: 246 bp; mutant: 150 bp): Primer 1 (wild type): 5-TTGCTAAAGCGCTACATAGGA-3′, Primer 2 (mutant): 5-CCTTCCTGAAGCAGTAGAGCA-3′, Primer 3 (common): 5-GCCTTATTGTGGAAGGACTG-3′.

### Longa score

Mice were scored behaviorally at different time points after ICH induction. The scores were calculated using the Longa method. No neurological deficit: 0 points; inability to fully extend the forepaws on the paralyzed side: 1 point; circling to the paralyzed side during walking: 2 points; dumping to the paralyzed side during walking: 3 points; inability to walk automatically, with loss of consciousness: 4 points.

### Statistical analysis

SPSS software (version 17.0) was used for the statistical analyses. All values for each group are presented as mean ± SD. Parametric and nonparametric tests were used according to the homogeneity of variance. According to different comparison situations, statistical differences were analyzed using Student’s *t*-test or one-way ANOVA, as appropriate, with Sidak’s or Turkey’s multiple comparisons test. *P* < 0.05 indicated that the difference was statistically significant.

## Results

### Homer1 expression peaked on the third day after ICH induction and colocalized with astrocytes

**Fig. 1:**
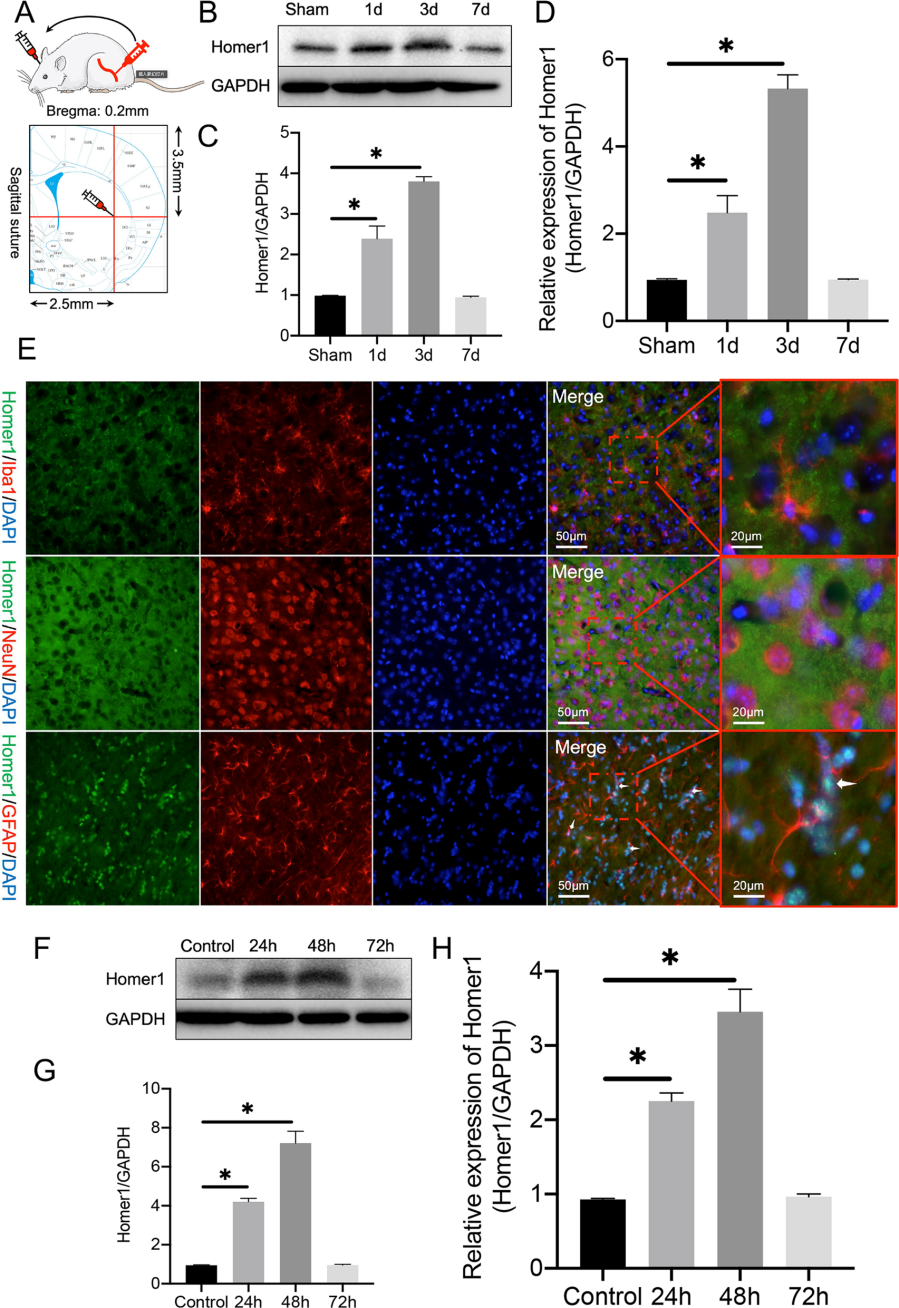
Protein and mRNA expression levels of Homer1 after ICH. **A** Schematic diagram of ICH modeling. **B** Homer1 protein expression in vivo after ICH. The blots are representative of other replicates in those groups. **C** Quantification of result in panel B [*F* (3, 16) = 283.4, *P* < 0.0001]. **D** Homer1 mRNA expression in vivo after ICH [*F* (3, 16) = 417.4 *P* < 0.0001].** E** Immunofluorescence was used to detect the co-localization of Homer1 with neurons, microglia, and astrocytes.** F** Expression of Homer1 protein in a cellular ICH model. The blots are representative of other replicates in those groups.** G** Quantification of result in panel *F* [*F* (3, 8) = 197.5, *P* < 0.0001]. **H** Expression of Homer1 mRNA in a cellular ICH model [*F* (3, 8) = 875.4, *P* < 0.0001]. The data were analyzed using one-way analysis of variance and all data are expressed as the mean 66262 ± standard deviation. *P < 0.05 represents a statistically significant difference between the two groups. Each experiment was repeated three times

First, according to a previously described method, autologous blood from the mouse femoral artery was extracted for intracranial injection to induce ICH [26] (Fig. 1A). The expression of Homer1 protein increased after ICH, peaked on the third day after the induction, and returned to the level of the sham group on the seventh day (Fig. 1B, C). The change in the trend of the *Homer1* transcription level was consistent with that of the protein level (Fig. 1D). After staining the brain tissue at the peak of Homer1 expression on the third day after ICH induction, we found that Homer1 was mainly located in astrocytes at the site of ICH (Fig. 1E). To further verify the expression of Homer1 in astrocytes, we extracted primary astrocytes from neonatal mice and treated them with erythrocyte lysate (1 μL of red blood cell lysate per mL of medium) to create an in vitro ICH inflammation model. After erythrocyte lysate treatment, the protein (Fig. 1F, G) and mRNA (Fig. 1H) levels of Homer1 initially increased and then decreased, and the expression level reached its peak 48 h after ICH induction.

### Simultaneous A1 astrocyte phenotype conversion and change of Homer1 expression after ICH induction in vivo and in vitro

**Fig. 2:**
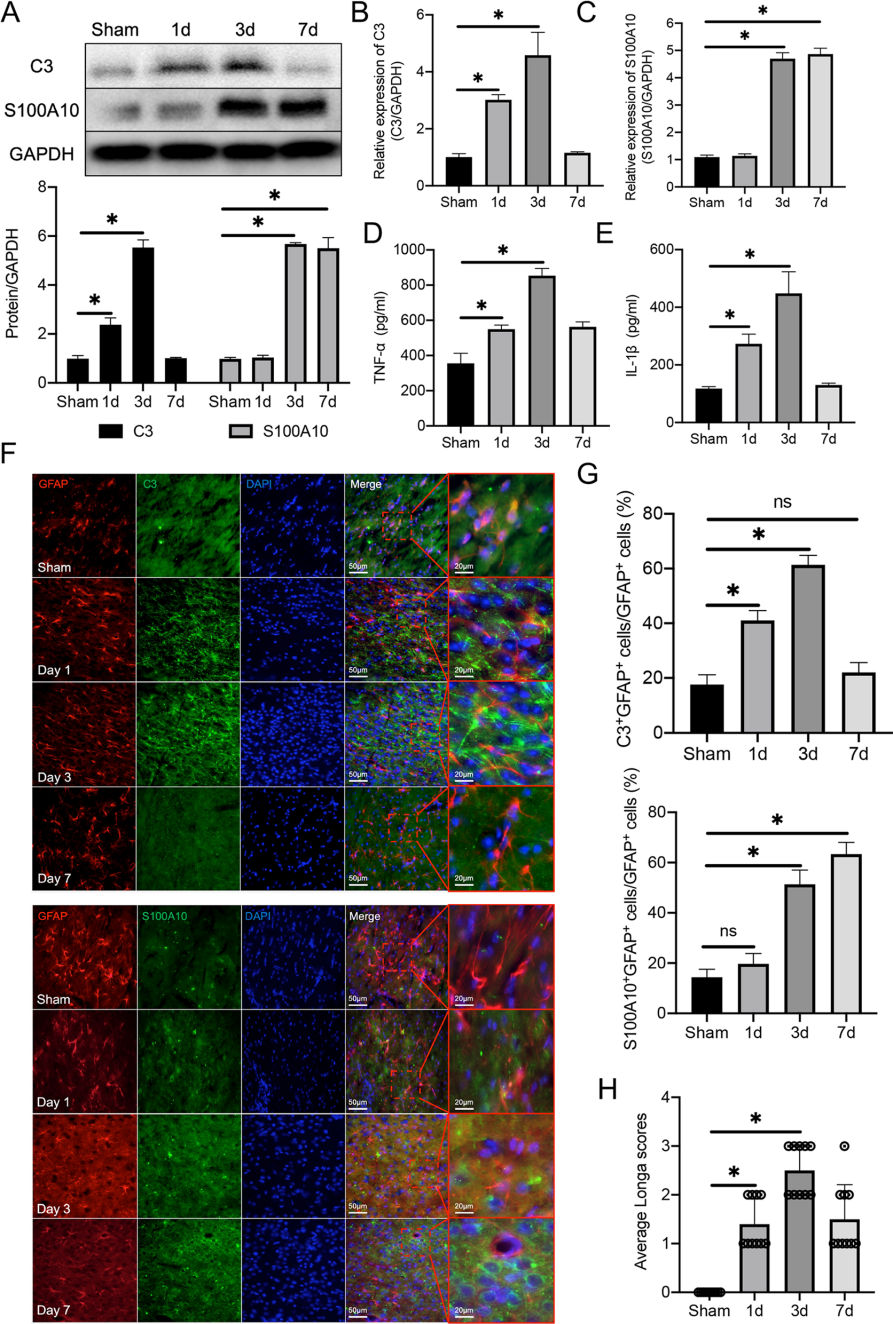
Transcription and translation levels of C3 and S100A10 after ICH in mice. **A** Protein expression of C3 and S100A10 in hemorrhagic tissues after ICH [C3: *F* (3, 16) = 509.4, *P* < 0.0001; S100A10: *F* (3, 16) = 936.0, *P* < 0.0001]. The blots are representative of other replicates in those groups.** B** mRNA level of C3 [*F* (3, 16) = 93.73, *P* < 0.0001].** C** mRNA level of S100A10 [*F* (3, 16) = 974.1, *P* < 0.0001).** D** expression of TNF-α [*F* (3, 16) = 133.8, *P* < 0.0001].** E** expression of IL-1β [*F* (3, 16) = 63.40, *P* = 0.0001].** F** Representative photographs of C3, S100A10 and GFAP co-staining of frozen sections of brain tissue at different time points after ICH.** G** Quantification of result in panel *F* [C3: *F* (3, 16) = 206.6, *P* < 0.0001; S100A10: *F* (3, 16) = 188.3, *P* < 0.0001].** H** Longa scores of mice at different time points after ICH [*F* (3, 36) = 40.47, *P* < 0.0001]. The data were analyzed using one-way analysis of variance and all data are expressed as the mean ± standard deviation. *P < 0.05 represents a statistically significant difference between the two groups. Each experiment was repeated three times

To detect changes in astrocytes after ICH, we detected the marker C3 in A1 phenotype astrocytes and S100A10 in A2 phenotype astrocytes. WB analysis showed that the expression of C3 gradually increased on the first and third day after ICH, and the expression of S100A10 gradually increased on the third and seventh days after ICH (Fig. 2A). The mRNA levels of *C3* (Fig. 2B) and *S100A10* (Fig. 2C) were consistent with the protein levels. A1 phenotype astrocytes also regulate the expression of classical inflammatory factors IL-1β and TNF-α. ELISA revealed that the expression of IL-1β and TNF-α in hemorrhagic brain tissue peaked on the third day, which was consistent with the expression trend of C3 (Fig. 2D, E). Meanwhile, immunohistochemical analysis of frozen tissue sections showed that the number of C3^+^GFAP^+^ cells was the largest on the third day after ICH induction, and the number of S100A10^+^GFAP^+^ cells increased on the third and seventh days after induction (Fig. 2F, G) (C3^+^GFAP^+^ cells: Sham vs. 1d vs. 3d vs. 7d: 18.40 ± 2.88 vs. 40.20 ± 2.864 vs. 61.40 ± 3.507 vs. 22.80 ± 2.864, respectively) (S100A10^+^GFAP^+^ cells: Sham vs. 1d vs. 3d vs. 7d: 13.60 ± 2.510 vs. 20.60 ± 3.286 vs. 51.00 ± 5.612 vs. 64.40 ± 3.715, respectively). In addition, the Longa score of ICH mice showed that hemiplegia was more serious on the third day (Fig. 2H) (Sham vs. 1d vs. 3d vs. 7d: 0.000 ± 0.000 vs. 1.400 ± 0.516 vs. 2.500 ± 0.527 vs. 1.500 ± 0.710, respectively).

**Fig. 3:**
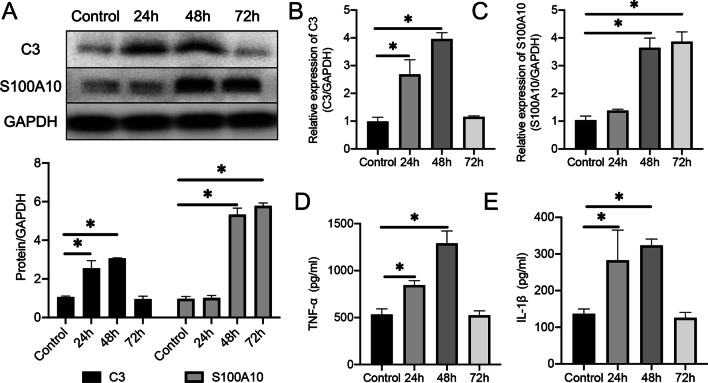
Transcription and translation levels of C3 and S100A10 after erythrocyte lysate stimulation in primary astrocytes. **A** Protein expression of C3 and S100A10 in primary astrocytes after erythrocyte lysate stimulation [C3: *F* (3, 8) = 74.4, *P* < 0.0001; S100A10: *F* (3, 8) = 519.8, *P* < 0.0001]. The blots are representative of other replicates in those groups.** B** mRNA level of C3 [*F* (3, 8) = 272.9, *P* < 0.0001).** C** mRNA level of S100A10 [*F* (3, 8) = 293, *P* < 0.0001).** D** expression of TNF-α [*F* (3, 8) = 37.69, *P* < 0.0001].** E** expression of IL-1β [*F* (3, 8) = 20.16, *P* = 0.0004]. The data were analyzed using one-way analysis of variance and all data are expressed as the mean ± standard deviation. *P < 0.05 represents a statistically significant difference between the two groups. Each experiment was repeated three times

In the primary astrocyte ICH model, WB (Fig. 3A) and qPCR (Fig. 3B, C) results showed that the expression of C3 began to increase after 24 h of erythrocyte lysate stimulation and reached its peak at 48 h, while the expression of S100A10 began to increase after 48 h and remained high until 72 h. TNF-α (Fig. 3D) and IL-1β (Fig. 3E) in the cell supernatant also began to increase after erythrocyte lysate stimulation and were most highly expressed 48 h after stimulation. Together with the in vivo results, these data illustrate that mice had the most severe inflammatory response on the third day after ICH, and the phenotype of astrocytes changed from A1 to A2.

### Homer1 promotes A1 astrocyte phenotype conversion in vivo and in vitro

**Fig. 4:**
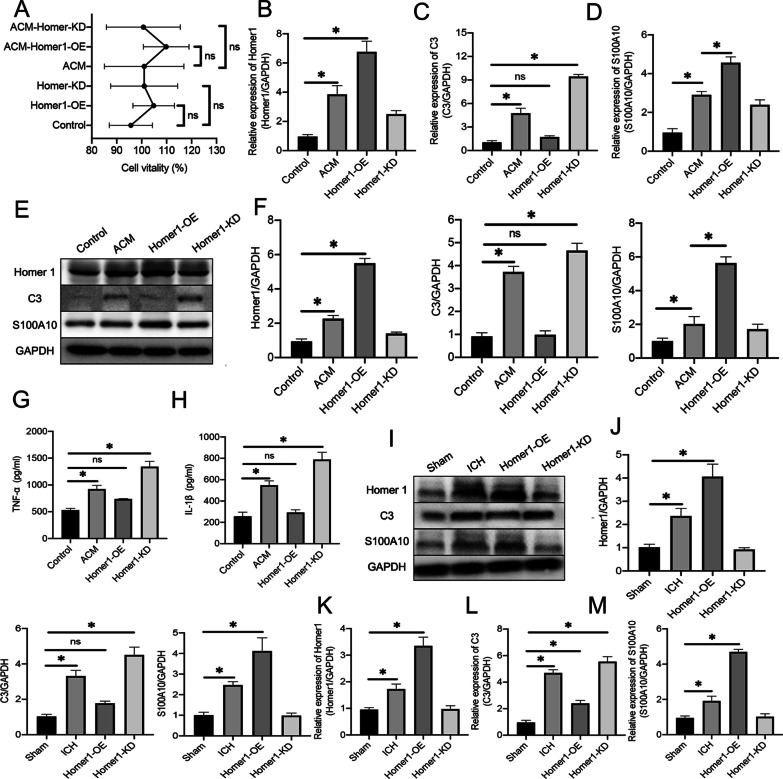
Effects of Homer1-OE and Homer1-KD on transcription and translation level of C3 and S100A10 in vivo and in vitro. **A** Effects of Homer1-OE and Homer1-KD on cell activity. the modified cells were replaced with ACM medium and cultured for 48 h to reach the most serious state of inflammation [normal medium: *F* (2, 6) = 1.760, *P* = 0.2504; ACM medium: *F* (2, 6) = 0.6375, *P* = 0.5610]. qPCR was used to detect the transcription levels of Homer1 (**B**) [*F* (3, 8) = 146.5, *P* < 0.0001], C3 (**C**) [*F* (3, 8) = 179.6, *P* < 0.0001) and S100A10 (**D**) [*F* (3, 8) = 196.7, *P* < 0.0001] in each group; WB was used to detect the translation level of Homer1, C3 and S100A10 (**E**) in each group and quantify the results (**F**) [Homer1: *F* (3, 8) = 334.0, *P* < 0.0001; C3: *F* (3, 8) = 136.7, *P* < 0.0001; S100A10: *F* (3, 8) = 146.1, *P* < 0.0001]; The blots are representative of other replicates in those groups. ELISA was used to detect the secretion levels of TNF-α (**G**) [*F* (3, 8) = 56.76, *P* < 0.0001] and IL-1β (**H**) [*F* (3, 8) = 140.1, *P* < 0.0001] in cell supernatant of each group. In vivo, WB was used to detect the translation levels of Homer1, C3 and S100A10 in the brain tissue of mice in each group on the 3rd day after ICH (**I**), and the results were quantified (**J**) [Homer1: *F* (3, 16) = 103.3, *P* < 0.0001; C3: *F* (3, 16) = 170.8, *P* < 0.0001; S100A10: *F* (3, 16) = 106.2, *P* < 0.0001]; The blots are representative of other replicates in those groups. qPCR was used to detect the transcriptional levels of Homer1 (**K**) [*F* (3, 16) = 157.3, *P* < 0.0001], C3 (**L**) [*F* (3, 16) = 389.2, *P* < 0.0001] and S100A10 (**M**) [*F* (3, 16) = 581.7, *P* < 0.0001] in the brain of mice 3 days after ICH. The data were analyzed using one-way analysis of variance and all data are expressed as the mean ± standard deviation. *P < 0.05 represents a statistically significant difference between the two groups. ns: no statistical difference. Each experiment was repeated three times

To investigate the effect of Homer1 on the phenotypic conversion of astrocytes, primary astrocytes were transduced with Homer1-OE and Homer1-KD using lentiviral transduction. In addition, we also cultured astrocytes in ACM in vitro to simulate the microenvironment dominated by astrocytes with the A1 phenotype on the third day after ICH in vivo. First, the results of the cell activity test showed that Homer1-OE and Homer1-KD had no effect on cell activity in either common medium or ACM (Fig. 4A). According to previous experimental results, the modified cells were recultured in ACM and cultured for 48 h to allow the cells to reach the most severe state of inflammation. qPCR (Fig. 4B–D) and WB (Fig. 4E, F) results showed that the expression of Homer1, C3, and S100A10 increased after astrocytes were cultured in ACM. In astrocytes cultured with ACM, Homer1-OE significantly decreased the expression of C3 and increased the expression of S100A10 while Homer1-KD increased C3 levels and decreased S100A10 levels. In addition, Homer1-OE decreased the release of TNF-α (Fig. 4G) and IL-1β (Fig. 4H), whereas Homer1-KD exacerbated its expression.

**Fig. 5:**
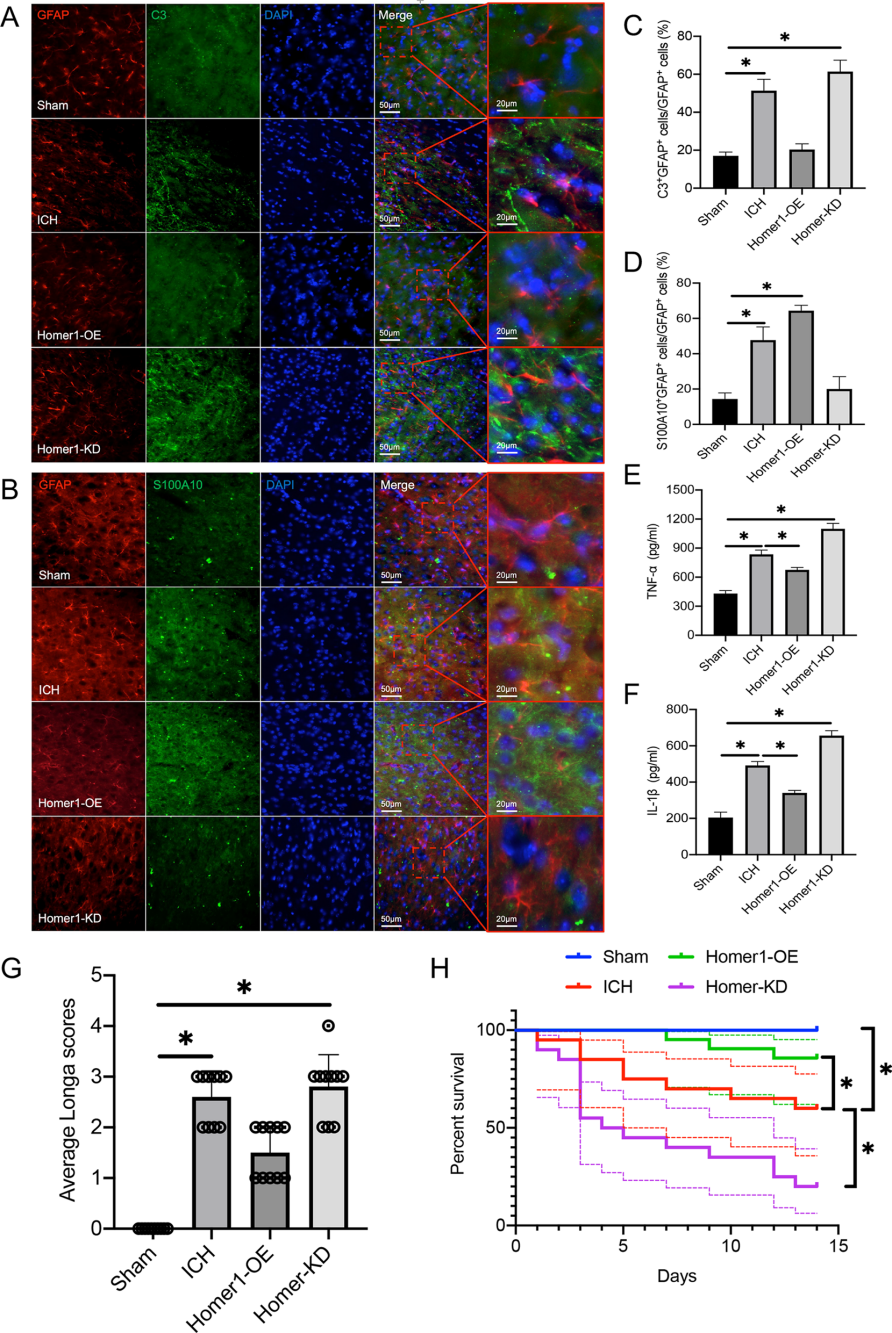
Homer1-OE increased the proportion of S100A10^+^GFAP^+^ cells and improved behavioral score and survival time. **A** Representative photographs of C3 and GFAP co-staining of frozen sections of brain tissue in different groups 3 days after intracerebral hemorrhage.** B** Representative photographs of S100A10 and GFAP co-staining of frozen sections of brain tissue in different groups 3 days after intracerebral hemorrhage.** C** Quantification of result in panel A [*F* (3, 16) = 167.3, *P* < 0.0001].** D** Quantification of result in panel B [*F* (3, 16) = 122.0, *P* < 0.0001].** E** expression of TNF-α [*F* (3, 16) = 223.6, *P* < 0.0001].** F** expression of IL-1β [*F* (3, 16) = 352.4, *P* < 0.0001]. **G** Longa scores of different groups on the 3rd day after ICH [*F* (3, 36) = 69.85, *P* < 0.0001].** H** Survival curve of mice in each group (*n* = 20) after ICH operation [log-rank (Mantel–Cox) test: Chi-square = 40.81; df = 3; *P* < 0.0001]. The data were analyzed using one-way analysis of variance and all data are expressed as the mean ± standard deviation. *P < 0.05 represents a statistically significant difference between the two groups

In vivo, we first used AAV to stably overexpress Homer1 or knock-down Homer1 at the ICH site in mice. The mouse ICH model was induced 3 weeks after AAV injection, and AAV regulation efficiency and molecular biological experiments were detected on the third day after ICH induction. Compared with ordinary ICH mice, Homer1-OE mice expressed more S100A10 in the brain at the transcriptional (Fig. 4K–M) and translational levels (Fig. 4I, J), whereas Homer1-KD mice expressed more C3. In addition, the results of immunohistochemistry experiments showed that the proportion of S100A10^+^GFAP^+^ cells in Homer1-OE mice was higher (Fig. 5B and D) (Sham vs. 1d vs. 3d vs. 7d: 14.40 ± 3.51 vs. 46.20 ± 6.34 vs. 65.00 ± 2.45 vs. 21.60 ± 5.46, respectively), while the proportion of C3^+^GFAP^+^ cells in Homer1-KD mice was higher (Fig. 5A and C) (Sham vs. 1d vs. 3d vs. 7d: 16.60 ± 1.67 vs. 50.20 ± 5.17 vs. 21.00 ± 2.45 vs. 62.60 ± 4.93, respectively). ELISA results of bleeding tissues also showed that Homer1-OE effectively reduced the expression of TNF-α (Fig. 5E) and IL-1β (Fig. 5F), whereas Homer1-KD exacerbated the inflammatory response. Finally, behavioral tests and survival analysis showed that Homer1-OE decreased the average Longa score of ICH mice (Fig. 5G) (Sham vs. 1d vs. 3d vs. 7d: 0.00 ± 0.00 vs. 2.60 ± 0.52 vs. 1.50 ± 0.53 vs. 2.80 ± 0.63) and prolonged the survival time of mice (Fig. 5H), while Homer1-KD had the opposite effect. These data illustrate that Homer1 plays a protective role by inducing a shift in astrocyte phenotype from A1 to A2 in ICH.

### Overexpression of Homer1 inhibited the activation of MAPK signaling

**Fig. 6:**
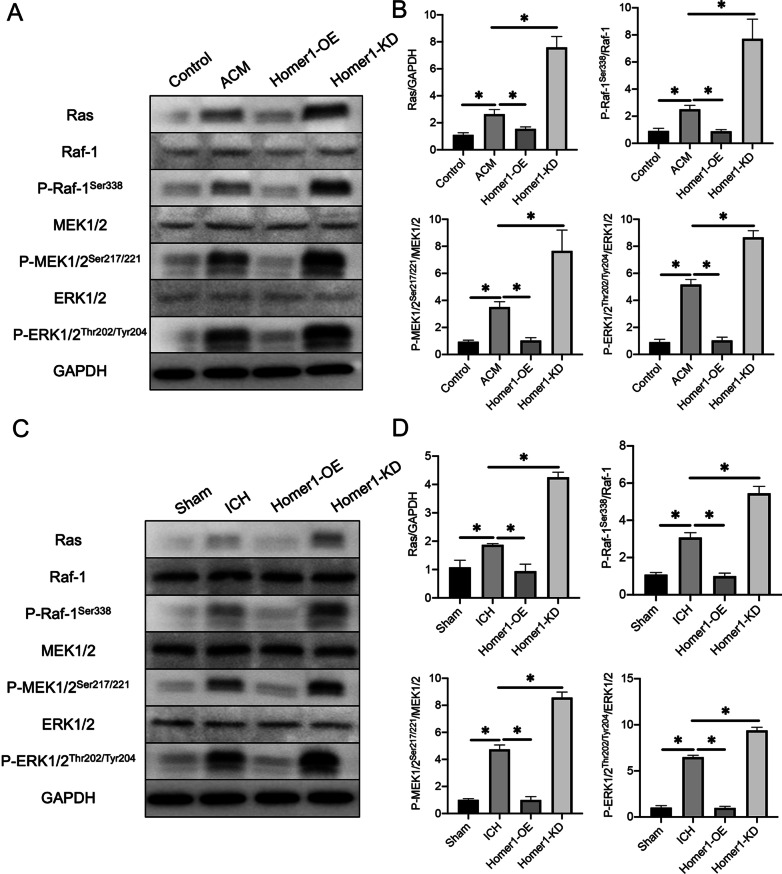
Effects of Home1-OE and Homer-KD on MAPK signaling. **A** WB was performed to examine the effect of Homer1-OE and Homer1-KD on MAPK signaling-related protein expression in primary astrocytes in vitro. The blots are representative of other replicates in those groups. **B** Quantification of result in panel A [Ras: *F* (3, 8) = 92.21, *P* < 0.0001; P-Raf-1^Ser338^/Raf-1: *F* (3, 8) = 57.06, *P* < 0.0001; P-MEK1/2/MEK1/2: *F* (3, 8) = 132.8, *P* < 0.0001; P-ERK1/2/ERK1/2: *F* (3, 8) = 278.4, *P* < 0.0001]. **C** WB was performed to examine the effect of Homer1-OE and Homer1-KD on MAPK signaling-related protein expression in cerebral hemorrhage site in vivo. The blots are representative of other replicates in those groups. **D** Quantification of result in panel C [Ras: *F* (3, 16) = 316.7, *P* < 0.0001; P-Raf-1^Ser338^/Raf-1: *F* (3, 16) = 376, *P* < 0.0001; P-MEK1/2/MEK1/2: *F* (3, 16) = 882.1, *P* < 0.0001; P-ERK1/2/ERK1/2: *F* (3, 16) = 1579, *P* < 0.0001]. The data were analyzed using one-way analysis of variance and all data are expressed as the mean ± standard deviation. *P < 0.05 represents a statistically significant difference between the two groups. Each experiment was repeated three times

The MAPK canonical signaling pathways include Ras, Raf-1, MEK1/2, and ERK1/2. While MAPK signaling plays an important role in tumor growth [28], the regulatory mechanisms of MAPK signaling and inflammation have been reported in an increasing number of studies [29, 30]. In addition, MAPK is an important initiator and modulator of astrocyte reactivity [31, 32]. To investigate whether Homer1 regulates inflammatory responses via MAPK signaling after ICH, we examined the expression of MAPK signaling-related proteins in in vivo and in vitro ICH models. WB results showed that after primary astrocytes were cultured in ACM for 48 h, the expression of Ras and the ratios of P-Raf-1^Ser338^ /Raf-1, P-MEK1/2/MEK1/2, and P-ERK1/2/ERK1/2 were increased. Homer1-OE inhibited the increase in protein expression induced by ACM, while Homer1-KD further promoted the increase in protein expression induced by ACM (Fig. 6A, B). Meanwhile, the results of animal experiments showed that MAPK signaling-related protein expression was significantly lower in mice with Homer1-OE than in the normal ICH group, whereas related protein expression was significantly higher in mice with Homer1-KD than in the ICH group (Fig. 6C, D). These data illustrate that Homer1 may play a protective role after ICH by inhibiting MAPK signaling.

### Homer1 protein and selumetinib, a non-ATP competitive MEK1/2 inhibitor, improve the outcome after ICH in Homer1^flox/flox^/Nestin-Cre^+/−^ mice

**Fig. 7:**
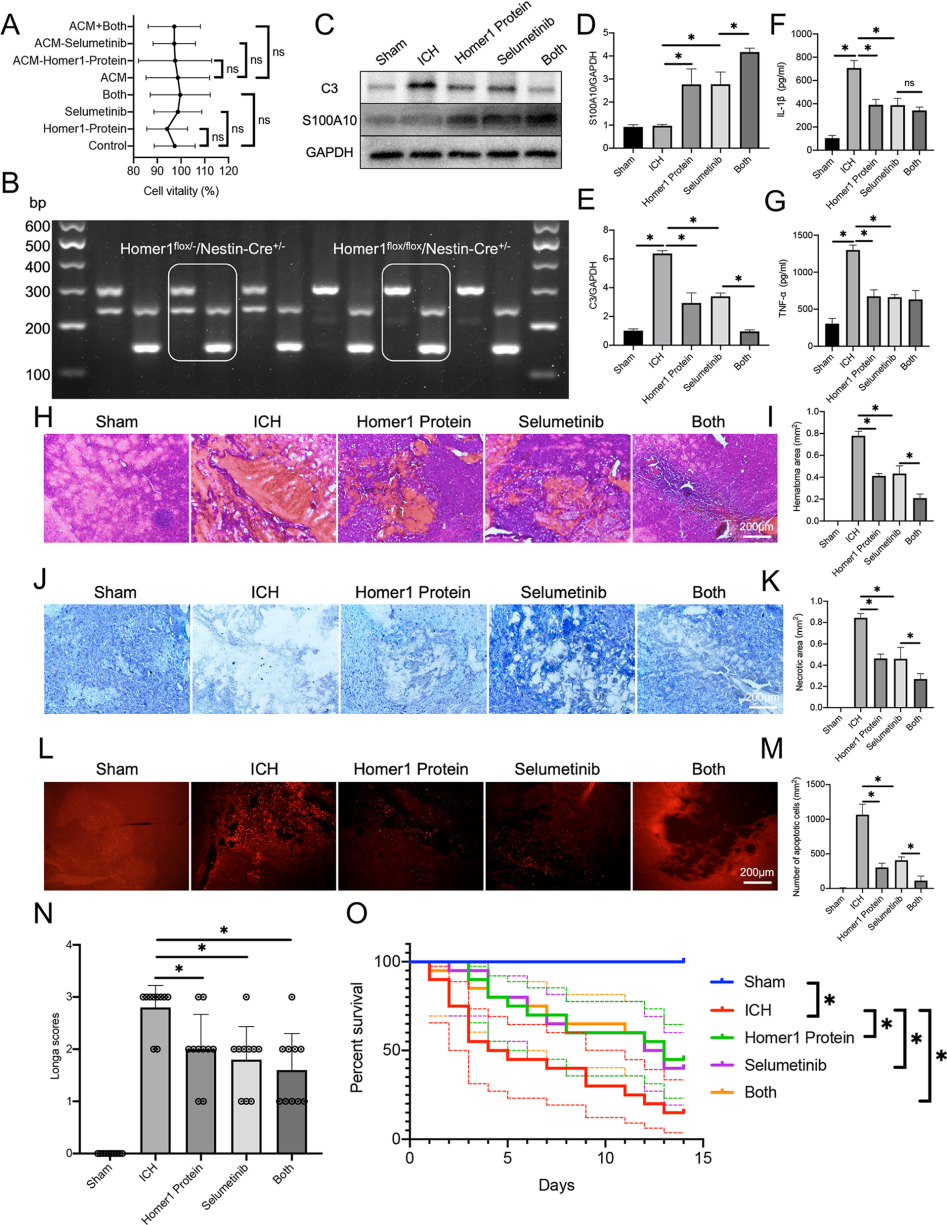
Homer1 protein and Selumetinib improve the pathological indexes and prognosis of ICH. **A** Effects of Homer1 protein and Selumetinib on cell activity [normal medium: *F* (3, 8) = 0.3731 *P* = 0.7748; ACM medium: *F* (3, 8) = 0.5067, *P* = 0.6885].** B** Genotype identification of Homer1^flox/flox^/Nestin-Cre^+/−^ mice.** C** WB was used to detect the translation levels of C3 and S100A10 in the brain tissue of mice in each group on the 3rd day after ICH and the results were quantified in **D** [*F* (4, 20) = 72.42, *P* < 0.0001] and **E** [*F* (4, 20) = 183.6, *P* < 0.0001]. The blots are representative of other replicates in those groups. **F** expression of IL-1β [*F* (4, 20) = 99.59, *P* < 0.0001]. **G** expression of TNF-α [*F* (4, 20) = 98.31, *P* < 0.0001].** H** Representative photographs of HE staining of brain tissue in each group.** I** Quantification of result in panel H [*F* (4, 20) = 279, *P* < 0.0001]. **J** Representative photographs of Nissl staining of brain tissue in each group.** K** Quantification of result in panel J [*F* (4, 20) = 196.6, *P* < 0.0001].** L** Representative photographs of TUNEL staining of brain tissue in each group.** M** Quantification of result in panel L [*F* (4, 20) = 159.6, *P* < 0.0001].** N** Longa scores of different groups on the 3rd day after ICH [*F* (4, 45) = 34.68, *P* < 0.0001].** O** Survival curve of mice in each group (*n* = 20) after ICH operation [Log-rank (Mantel–Cox) test: Chi-square = 21.4; df = 4; *P* = 0.0003]. The data were analyzed using one-way analysis of variance and all data are expressed as the mean ± standard deviation. *P < 0.05 represents a statistically significant difference between the two groups. *ns* no statistical difference

The results of previous experiments suggest that Homer1 plays a protective role in ICH by affecting the MAPK signaling pathway. Therefore, we chose Homer1 and selumetinib, a non-competitive ATP MEK1/2 inhibitor, to explore effective treatments for ICH. Astrocytes were cultured in normal DMEM and ACM and the medium was supplemented with 1 μM Homer1 protein or selumetinib, and cell viability was measured after 48 h of culture. The results showed that Homer1 protein and selumetinib did not affect the activity of primary astrocytes (Fig. 7A). To better reflect the role of Homer1 in ICH, conditional knockout mice were generated. Genotypes (Homer1^flox/flox^/Nestin-Cre^+/−^) were identified by PCR (Fig. 7B). Mice were used to create ICH models and after in situ injection of Homer1 protein (5 mg/kg) and selumetinib (5 mg/kg), mouse brain tissues were collected for molecular biological and pathological examination on the third day after ICH. Compared to the ICH model of normal mice (Fig. 2B), the expression of C3 in Homer1^flox/flox^/Nestin-Cre^+/−^ mice was higher on the third day after ICH. In addition, compared with the ICH group, the expression of C3 in the brain tissue decreased significantly, and the expression of S100A10 increased after injection of Homer1 protein or selumetinib. The effect of the combined administration of Homer1 protein and selumetinib was more obvious than that of the injection of each drug individually (Fig. 7C–E). The results of ELISA assays showed that both drugs reduced the expression of IL-1β and TNF-α in the brain (Fig. 7F, G). Additionally, mouse brain tissue was used to prepare frozen sections for pathological detection. HE staining showed that Homer1 protein and selumetinib injection reduced the bleeding area of brain tissue (Fig. 7H, I) (Sham vs. ICH vs. Homer1 protein vs. Selumetinib vs. Both: 0.00 ± 0.00 vs. 0.77 ± 0.03 vs. 0.41 ± 0.02 vs. 0.44 ± 0.07 vs. 0.22 ± 0.23, respectively). Nissl staining showed that Homer1 protein and selumetinib injection improved the activity of neurons at the bleeding site (Fig. 7J, K) (Sham vs. ICH vs. Homer1 protein vs. Selumetinib vs. Both: 0.00 ± 0.00 vs. 0.85 ± 0.04 vs. 0.47 ± 0.04 vs. 0.48 ± 0.09 vs. 0.28 ± 0.04, respectively), while TUNEL staining showed that Homer1 protein and selumetinib injection significantly reduced the number of apoptotic cells in brain tissue (Fig. 7L, M) (Sham vs. ICH vs. Homer1 protein vs. Selumetinib vs. Both: 5.60 ± 3.71 vs. 1039 ± 134.6 vs. 293.8 ± 55.86 vs. 398.2 ± 41.84 vs. 98.20 ± 53.49, respectively). Finally, we found that the drugs effectively reduced the Longa score (Fig. 7N) (Sham vs. ICH vs. Homer1 protein vs. Selumetinib vs. Both: 0.00 ± 0.00 vs. 2.80 ± 0.42 vs. 2.00 ± 0.67 vs. 1.80 ± 0.63 vs. 1.60 ± 0.70, respectively) on the third day after ICH and significantly prolonged the survival time of mice after ICH (Fig. 7O).

### The efficacy of Homer1 in the treatment for ICH is associated with reduced expression of the inflammatory factor TNFSF10 and increased expression of the anti-inflammatory factors activin A, persephin, and TWEAK

**Fig. 8:**
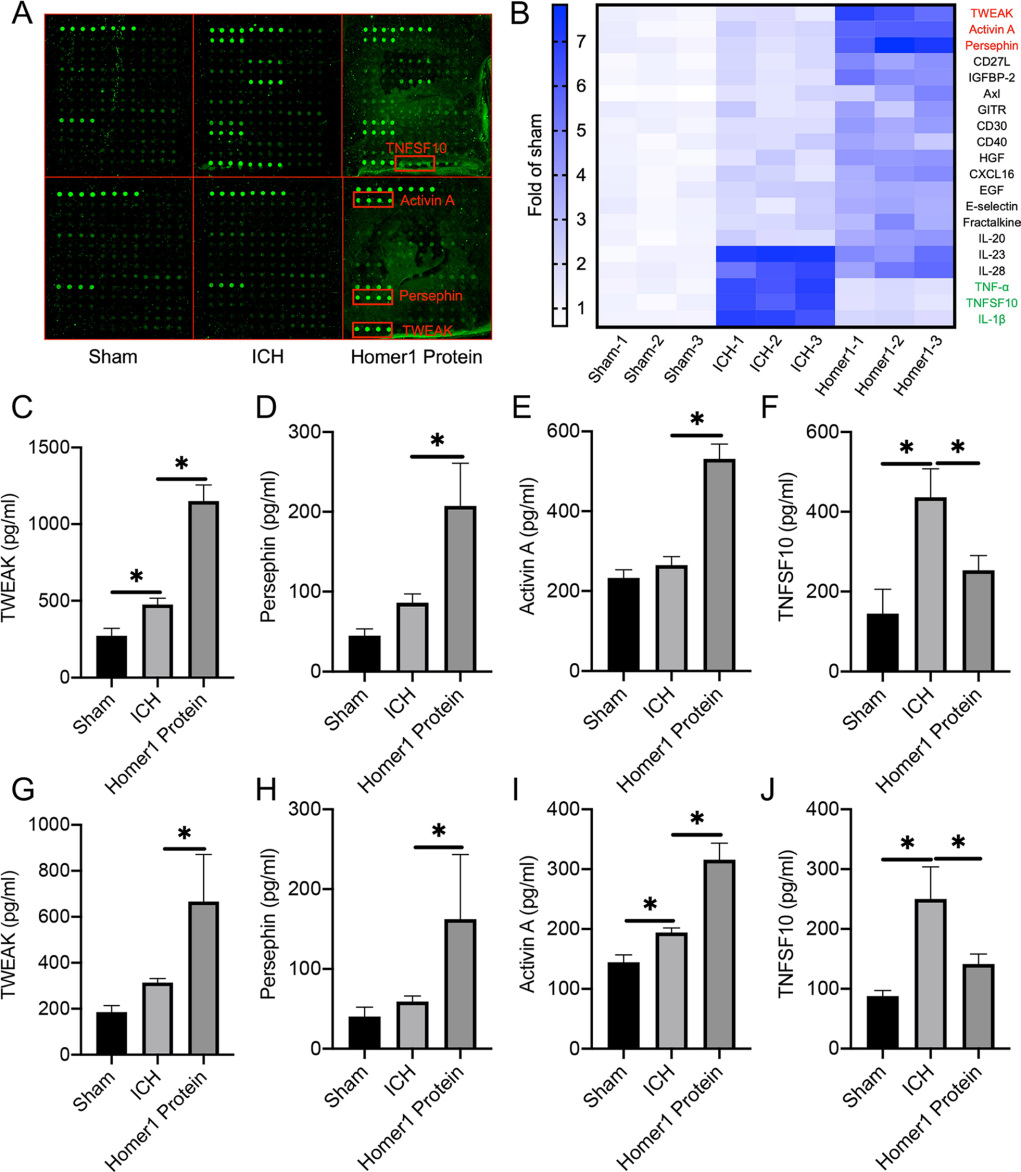
The inflammatory indexes in brain tissue and cerebrospinal fluid were detected by protein chip. **A** Representative experimental diagram of chip slide carrier. **B** Heat map of protein chip results. **C** Expression level of TWEAK in hemorrhagic tissue [*F* (2, 6) = 125.5, *P* < 0.0001]. **D** Expression level of Persephin in hemorrhagic tissue [*F* (2, 6) = 20.94, *P* = 0.002]. **E** Expression level of Activin A in hemorrhagic tissue [*F* (2, 6) = 107.2, *P* < 0.0001]. **F** Expression level of TNFSF10 in hemorrhagic tissue [*F* (2, 6) = 19.22, *P* = 0.0025]. **G** Expression level of TWEAK in CSF [*F* (2, 6) = 12.99, *P* = 0.0066]. **H** Expression level of Persephin in CSF [*F* (2, 6) = 5.736, *P* = 0.0405]. **I** Expression level of Activin A in CSF [*F* (2, 6) = 70.95, *P* < 0.0001]. **J** Expression level of TNFSF10 in CSF [*F* (2, 6) = 18.82, *P* = 0.0026]. The data were analyzed using one-way analysis of variance and all data are expressed as the mean ± standard deviation. *P < 0.05 represents a statistically significant difference between the two groups

To study the detailed mechanism of Homer1 protein in the treatment of ICH, hemorrhagic brain tissues of the Sham group, ICH group, and Homer1^flox/flox^/Nestin-Cre^+/−^ mice injected with Homer1 protein were used for the detection of 200 index protein chips. The protein with the most obvious multiple changes between the ICH and Homer1 protein treatment groups was selected for further analysis (Fig. 8A, B). We found that compared with the ICH group, the expression levels of TNF-related weak inducer of apoptosis (TWEAK) (Fig. 8C), persephin (Fig. 8D), and activin A (Fig. 8E) in the Homer1 protein treatment group increased significantly, whereas the expression of TNF-α, tumor necrosis factor ligand superfamily member 10 (TNFSF10) (Fig. 8F), and IL-1β decreased significantly. However, using hemorrhagic brain tissue for clinical molecular detection is inconvenient. To verify whether the changes in these molecules specifically reflect the effectiveness of Homer1 protein in the treatment of ICH, we extracted the cerebrospinal fluid of mice for ELISA detection. The expression of TWEAK (Fig. 8G), persephin (Fig. 8H), and activin A (Fig. 8I) was upregulated after Homer1 protein treatment, while the expression of TNFSF10 (Fig. 8J) was downregulated. These data suggest that the mechanism of action of Homer1 protein in the treatment of ICH may be related to TWEAK, activin A, TNFSF10, and persephin. The expression changes of these proteins reflect the effectiveness of Homer1 protein in the treatment of ICH.

## Discussion

Our study indicated that Homer1 expression peaked on the third day after ICH and colocalized with astrocytes. Homer1 promotes A1 to A2 phenotypic conversion in astrocytes in vivo and in vitro. Overexpression of Homer1 inhibited the activation of MAPK signaling, whereas Homer1 knock-down increased the expression of pathway-related proteins. Homer1 protein and selumetinib, a non-ATP competitive MEK1/2 inhibitor, improved the outcome in Homer1^flox/flox^/Nestin-Cre^+/−^ mice after ICH. The efficacy of Homer1 in the treatment of ICH was associated with the reduced expression of TNFSF10 and enhanced expression of activin A, persephin, and TWEAK.

The Homer1 gene encodes a short isoform (Homer1a, aa1-186) and two long isoforms (Homer1b, aa1-354 and Homer1c, aa1-366). Homer1a lacks a coiled-coil domain and a leucine zipper, which antagonizes the multimerization of Homer and results in the disassembly of signal transduction protein complexes [33]. We have previously shown that Homer1a alleviates mitochondrial stress caused by endoplasmic reticulum stress after ischemia–reperfusion injury by inhibiting the PERK pathway [34]. Homer1a attenuates hydrogen peroxide-induced oxidative damage in HT-22 cells via AMPK-dependent autophagy [35]. Upregulation of Homer1a promotes retinal ganglion cell survival after retinal ischemia and reperfusion by interacting with the ERK pathway [36]. In this study, we found that Homer1 inhibited the phenotypic conversion of astrocytes by inhibiting the MAPK signaling pathway to improve outcome in mice after ICH. However, whether the neuroprotective effect of Homer1 after ICH is due to the short isotype Homer1a remains unclear and needs to be further clarified.

Clinically, conservative treatment or craniotomy is usually performed based on the amount of bleeding after ICH [37]. Antihypertensive drugs, hemostatic drugs, and mannitol are generally used for conservative treatment of ICH. In many animal studies, drugs developed for the treatment of ICH are administered via intraperitoneal injection [38–41]. While this route of administration is already unsuitable for therapy in human patients, many drugs also do not cross the blood–brain barrier, which limits the efficacy of the drugs. Intraoperative in situ administration appears to be more practical and convenient for the treatment of ICH. Our study found that when Homer1 protein was injected in situ into the bleeding site 10 min after ICH, the pathological indices could be effectively improved. Treatment with the Homer1 protein and MAPK inhibitor selumetinib effectively reduced apoptosis in brain tissue at the bleeding site and significantly prolonged the survival time of Homer1^flox/flox^/Nestin-Cre^+/−^ mice within 15 days (test it for up to 15 days). Although it is not necessary for the drug to pass through the blood–brain barrier after in situ injection, possible toxic effects of Homer1 and selumetinib on brain tissue should be taken into consideration. Fortunately, Homer1 protein and selumetinib did not affect the activity of primary astrocytes, and toxicological effects on nervous cells were not detected by HE staining and Nissl staining.

Although we found effective ways and drugs to be used after ICH, our study has some limitations. Because there is no commercial mouse Homer1 protein, the human Homer1 protein was used in this experiment. In fact, immune rejection caused by different species should also be considered, and the specific mechanisms and experimental results need to be further clarified. In addition, the degradation of the drugs after their injection into the brain needs to be considered. Both the Homer1 protein and MAPK inhibitor selumetinib are degraded in a complex chemical reaction. Although we observed that the pathology of ICH improved after injection of these two drugs, whether the degradation products play a role in this improvement remains to be tested. Our study identified a new mechanism and showed that Homer1 promotes the conversion of astrocytes through the MAPK pathway to improve the inflammatory response induced by ICH. Therefore, any modulation of this mechanism might have a therapeutic effect in ICH. A recent report suggested that C3 is more likely to be inhibited by the C3 receptor antagonist trifluoroacetate [42]. Astrocyte conversion after stroke has also been achieved using genetic methods [43]. Therefore, in addition to conventional pathway inhibitors, pharmacological antagonists and novel direct reprogramming of astrocytes may be used to explore the effect of Homer1 on the treatment of ICH. Finally, like stroke, aging is the most important non-modifiable risk factor in the incidence of exogenous or endogenous ICH [44]. To better transform our research into clinical practice, it should also be clarified whether the efficacy of Homer1 in the treatment of ICH is affected by age.

In our study, a mouse cytokine microarray showed that in addition to IL-1β and TNF-α, Homer1 protein treatment effectively reduced the expression of TNFSF10 (TRAIL). TNFSF10 induces inflammatory responses in human adipocytes, regulates the apoptosis of inflammatory neutrophils, and enhances the regression of inflammation [45]. In the present study, we found that the expression of TWEAK, activin A, and persephin was upregulated after Homer1 protein treatment. TWEAK, which is commonly involved in immune regulation, inflammation, and apoptotic processes, is a member of the TNF superfamily of cytokines [46, 47] and it has been shown that crosstalk between IFN-γ and TWEAK amplifies skin inflammation in psoriasis through miR-149 [48]. Activin A is believed to be a pleiotropic cytokine with pro-inflammatory and anti-inflammatory properties [49]. In contrast, persephin is a neurotrophic factor belonging to the GDNF family that promotes neuronal survival and prevents neuronal degeneration associated with injury, toxin exposure, or neurodegenerative diseases [50, 51]. Therefore, we suggest that the downregulation of the inflammation-inducing factor TNFSF10, upregulation of the neurotrophic factor persephin, and the coordination of activin A in pro-inflammatory and anti-inflammatory activity may be the relevant mechanisms by which the Homer1 protein exerts its therapeutic effects. Although TWEAK as a pro-inflammatory factor increased after Homer1 protein treatment, a close relationship between TWEAK and TNFSF10 was identified in a study by Prigozhina et al. in which Fn14·TRAIL was converted into a highly effective TRAIL oligomer upon binding to TWEAK [52]. Therefore, we hypothesize that Homer1 may also regulate inflammation after ICH through a complex regulatory mechanism involving TWEAK and TNFSF10, but the specific mechanism requires further studies.

## Conclusions

In conclusion, our study showed that Homer1 overexpression promotes the conversion of the astrocyte phenotype from A1 to A2 after ICH, both in vivo and in vitro, thereby inhibiting the ICH-induced inflammatory response. It is implied that in patients with ICH in clinical practice, the administration of Homer1 protein and MAPK inhibition may reduce the inflammatory response to reduce apoptosis of cells in the brain and improve the outcomes in patients with ICH.

## Data Availability

The datasets used and/or analyzed during the current study are available from the corresponding author on reasonable request.

## Abbreviations

CNS: Central nervous system
ICH: Intracerebral hemorrhage
Homer1: Homer scaffold protein 1
MAPK: Mitogen-activated protein kinase
OE: Overexpression
KD: Knockdown
qPCR: Quantitative real-time polymerase chain reaction
WB: Western blot
ACM: Astrocyte conditional medium
FBS: Fetal bovine serum
TWEAK: TNF-related weak inducer of apoptosis
TNFSF10: Tumor necrosis factor ligand superfamily member 10
AAV: Adeno-associated virus
